# Crustacean Hyperglycemic Hormone (cHH) as a Modulator of Aggression in Crustacean Decapods

**DOI:** 10.1371/journal.pone.0050047

**Published:** 2012-11-16

**Authors:** Laura Aquiloni, Piero G. Giulianini, Alessandro Mosco, Corrado Guarnaccia, Enrico Ferrero, Francesca Gherardi

**Affiliations:** 1 Dipartimento di Biologia Evoluzionistica “Leo Pardi”, Università degli Studi di Firenze, Firenze, Italy; 2 Dipartimento di Scienze della Vita, Università degli Studi di Trieste, Trieste, Italy; 3 International Centre for Genetic Engineering and Biotechnology, AREA Science Park, Trieste, Italy; Utrecht University, The Netherlands

## Abstract

Biogenic amines, particularly serotonin, are recognised to play an important role in controlling the aggression of invertebrates, whereas the effect of neurohormones is still underexplored. The crustacean Hyperglycemic Hormone (cHH) is a multifunctional member of the eyestalk neuropeptide family. We expect that this neuropeptide influences aggression either directly, by controlling its expression, or indirectly, by mobilizing the energetic stores needed for the increased activity of an animal. Our study aims at testing such an influence and the possible reversion of hierarchies in the red swamp crayfish, *Procambarus clarkii*, as a model organism. Three types of pairs of similarly sized males were formed: (1) ‘control pairs’ (CP, n = 8): both individuals were injected with a phosphate saline solution (PBS); (2) ‘reinforced pairs’ (RP, n = 9): the alpha alone was injected with native cHH, and the beta with PBS; (3) ‘inverted pairs’ (IP, n = 9): the opposite of (2). We found that, independently of the crayfish’s prior social experience, cHH injections induced (i) the expression of dominance behaviour, (ii) higher glycemic levels, and (iii) lower time spent motionless. In CP and RP, fight intensity decreased with the establishment of dominance. On the contrary, in IP, betas became increasingly likely to initiate and escalate fights and, consequently, increased their dominance till a temporary reversal of the hierarchy. Our results demonstrate, for the first time, that, similarly to serotonin, cHH enhances individual aggression, up to reverse, although transitorily, the hierarchical rank. New research perspectives are thus opened in our intriguing effort of understanding the role of cHH in the modulation of agonistic behaviour in crustaceans.

## Introduction

Given the nearly ubiquity of social hierarchy across animal species (reviewed in [1]), an intriguing purpose of behavioural studies is to understand how adaptive mechanisms underlay the formation and maintenance of social hierarchies and which chemical substances are involved in aggression.

Crayfish are excellent model organisms to study the proximate mechanisms that invertebrates adopt to establish and maintain dominance hierarchies [2]. They exhibit easily identifiable behavioural patterns that escalate giving rise to fights of increased severity until dominance hierarchies are formed [3]–[7]. At that point, the number and intensity of fights decrease and crayfish behave consistently with the social status achieved: the dominant displays raised postures, is the initiator of most attacks and gains first access to limited resources, whereas the subordinate individual displays submissive postures, escapes from the dominant’s attacks, and has limited access to resources [8]–[9].

The maintenance of stable hierarchies is certainly adaptive [10], since both fighting costs and risks of injuries are reduced [10]–[11], but the mechanisms underlying dominance stability is still under debate. As suggested by [12], the formation of stable dominance relationships in crayfish is driven by extrinsic factors (e.g. previous history and communication) and intrinsic chemical processes (e.g. the neurochemical state). Hence, a deeper understanding of the role of neuropeptides in triggering agonistic behaviour is crucial to solve the still heated debate around the mechanisms that maintain hierarchies.

To date, serotonin (5-HT, 5-hydroxytryptamine) is the main neuromodulator recognized to play an important role in controlling aggression in several crustacean decapods (the lobster *Homarus americanus*, [13]–[15]; the shore crab *Carcinus maenas*, [16]; the crab *Chasmagnathus granulatus*, [17]; the squat lobster *Munida quadrispina*, [18]), crayfish included (*Procambarus clarkii*, [19]; *Astacus astacus*, [20]–[21]). In particular, if compared to subordinate individuals, serotonin-treated crayfish fight for longer and more strongly, and less often retreat [20]–[21] [15] [19]. Serotonin has also a marked effect in elevating glucose level in hemolymph, as an adaptive response to the forthcoming fights [22]. Hyperglycaemia results from the mobilization of glycogen in target tissues (e.g. midgut glands and abdominal muscles), due to the activation of phosphorylase and the inhibition of glycogen synthase via the crustacean Hyperglycemic Hormone (cHH) [23].

Crustacean HH is a member of a family of eyestalk neuropeptides [24]–[25], which includes the Moult Inhibiting Hormone (MIH) and the Gonad Inhibiting Hormone (GIH): the cHH/MIH/GIH family. These neuropeptides are released through exocytosis from the sinus gland (SG), a neurohemal organ located in the eyestalk of decapod crustaceans. The main function of cHH is the regulation of glucose levels in the hemolymph. It is also involved in reproduction [26]–[27], moulting [28]–[29], lipid metabolism [30], and stress responses [31]–[33]. About 80 cHH-superfamily peptides have thus far been fully identified from several crustacean species.

Notwithstanding the high number of papers on the cHH chemical nature, studies on its biological activity remain still scanty [34] and, up to date, no data from the literature are available on the putative role of cHH as a modulator of aggression.

To fill this gap in knowledge, here we investigate the possible influence that cHH exerts on the agonistic behaviour of the red swamp crayfish, *Procambarus clarkii.* Specifically, we hypothesized that cHH, similarly to serotonin, could affect crayfish behaviour to the extent of reversing the hierarchical rank in combating pairs. To test this hypothesis, we manipulated the agonistic level of males in size-matched pairs through the injection of a dose of native cHH or phosphate saline solution (PBS) into the crayfish circulation. Our aims were to (1) describe the possible effect of cHH on the agonistic behaviour of crayfish and its duration, (2) assess the increased glycaemic level due to cHH injections, and (3) test whether possible changes in aggression associated with cHH injections are sufficient to reverse an established dominance hierarchy. Our general purpose is to quantify the possible effects of cHH on crayfish agonistic behaviour and to discuss the relative importance of other intrinsic/extrinsic factors in maintaining dominance hierarchies.

## Materials and Methods

### Collection and Holding Conditions

About 200 male crayfish were collected using baited traps from Lake Trasimeno (Umbria, central Italy) in July 2011 by professional fishermen. Once in the laboratory, each crayfish was individually marked onto its carapace with a waterproof paint and its cephalothorax length (from the tip of the rostrum to the posterior edge of the carapace) was measured using a vernier calliper (accuracy: ±0.1 mm).

Crayfish were kept for at least two weeks at the density of 15 m^−2^ in plastic tanks (80×60×60 cm) containing clay pots in excess as shelter and at a natural light-dark cycle at room temperature (24°C). They were fed *ad libitum* with live *Calliphora* sp. larvae. Water was changed weekly.

### Criteria for Choosing Experimental Crayfish

Only hard-shelled, intact, and sexually mature males were used for the experiment. A total of 80 individuals (cephalothorax length: 47.5±0.6 mm) were thus selected: 20 for the extraction of cHH and 60 for behavioural observations. Since dominance increases with body size in crayfish [3], the experimental pairs of fighting males were size matched (maximum difference in cephalothorax length: ±2 mm) to eliminate any factor that could induce an obvious bias to our experiments. Before the beginning of the experiment, crayfish were kept in isolation in opaque plastic aquaria (25×15×25 cm) for at least two weeks, which is a sufficient time to reset any previous social experience [35]. In no case did the crayfish meet each other prior to the experiment, so any effect of previous social experience can be excluded [36]. All crayfish were used only once to avoid pseudo-replication.

### Extraction of Native cHH

**Figure 1:**
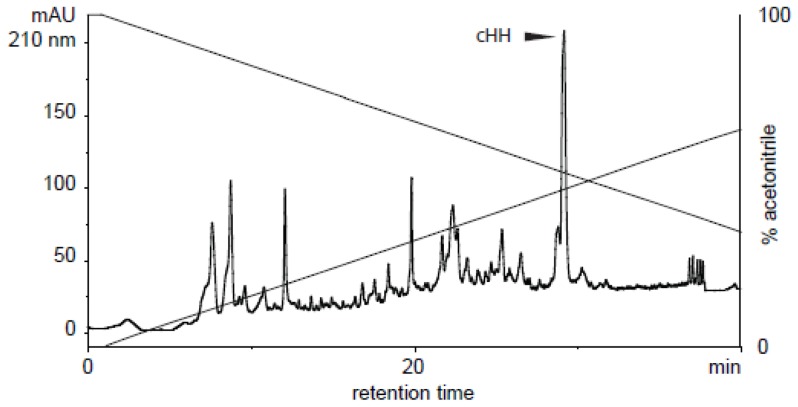
RP-HPLC profile of the crude extract of sinus glands. Mobile Phase A: 0.1% TFA in water. Mobile Phase B: 0.1% TFA in acetonitrile. Gradient: 0–100% B over 60 min at 1 mL min^−1^. Column: Zorbax SB-C18 4.6 × 150 mm.

Twenty animals were anesthetized for 5 min on ice before eyestalk ablation. From 40 eyestalks the crude extract of dissected sinus glands was collected by adding 200 µL of extraction solution (90% MetOH, 9% acetic acid, 1% H_2_O). After sonication, the sample was centrifuged at 12 000× g for 10 min at 4°C and the supernatant was collected. The pellet was suspended in 200 µL of the extraction solution, sonicated and centrifuged again, and the two supernatants were mixed together. The extract was purified on an RP-HPLC system (Gilson) equipped with a Zorbax SB-C18 4.6×150 mm column from Agilent Technologies Inc. (DE, USA) thermostated at 25°C. Mobile phase A was 0.1% TFA in water, mobile phase B was 0.1% TFA in acetonitrile. The separation was done using a gradient of 0–100% B in 60 min at 1 mL/min. The resulting chromatogram is shown in Figure 1.

The collected fractions were analyzed on an API150EX single quadrupole mass spectrometer (ABSciex), and those fractions containing the expected molecular mass of 8386 Da [37] were pooled and lyophilized. Peptide concentration was determined by UV absorbance at 280 nm using calculated ε values of 9315 M^−1^ cm^−1^ for the peptide oxidized form. The extinction coefficient was computed using the ProtParam programme on the ExPASy server [38].

### Experimental Design (Fig. 2)

Behavioural experiments were conducted in the laboratory from 0800 to 1400 h during August 2011 to reduce possible interference due to circadian changes in blood glucose level [39]. During observations, we recorded the effects through time of the injected native cHH extract on crayfish behaviour and examined whether such extract might induce a change in the hierarchy. The experiment was planned in five phases in sequence, as described below.

**Figure 2:**
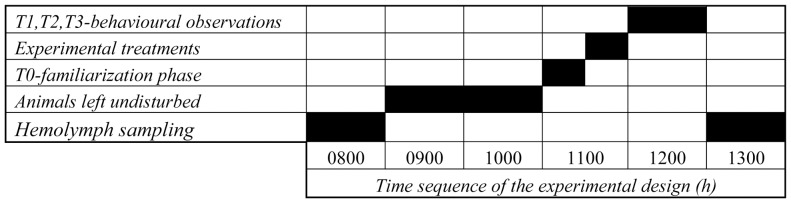
Horizontal time sequence of experimental design.

#### Phase 1: Hemolymph sampling and determination of initial glycemia

The animals were blotted dry and hemolymph (about 50 µl) was drawn from the pericardial sinus into sterile 1 mL syringes fitted with 25 g needles. All the animals were bled between 0800 and 0900 h and left undisturbed for 2 h. The sample was preserved on ice for 5 min to avoid coagulation and then centrifuged at 12 000× g for 2 min at 4°C to pellet the hemocytes. The supernatant was then collected. Glucose concentration (mg dL^−1^) in the hemolymph was assessed using the glucose oxidase method of a commercial kit (Hospitex Diagnostics).

#### Phase 2: Familiarization (T0)

The two opponents were kept in an experimental aquarium (a circular opaque PVC container, diameter: 30 cm) separated by an opaque PVC divider for 10-min acclimatization. The familiarization started with the removal of the divider and lasted 20 min (T0), during which time crayfish behaviour was recorded by a digital camera (Samsung VP-L800) for subsequent blind analysis (see below). Simultaneously, an experienced observer (LA) recorded the winner of each fight so that, at the end of familiarization, we could determine the dominant –alpha crayfish (and consequently the subordinate –beta crayfish) for each pair, that is, the winner (and the loser) of more than 60% of the total fights [40]. The ‘winner’ was defined as the crayfish that did not retreat or that retreated after the opponent showed a motionless posture, which is typical of a subordinate [4]. Trials where dominance was not clearly established were excluded from the analysis. A total of 26 (out of 30) size-matched pairs were observed and alpha and beta crayfish were assessed.

#### Phase 3: Experimental treatments

The above selected pairs were randomly assigned to one of the following treatments: (1) ‘control pairs’ (CP, n = 8): both males were injected with 100 µL of phosphate buffered saline (PBS); (2) ‘reinforced pairs’ (RP, n = 9): the alpha was injected with 100 µL of native cHH solution, and the beta with 100 µL of PBS; (3) ‘inverted pairs’ (IP, n = 9): the opposite of the previous treatment.

To obtain cHH solutions, lyophilised native cHH was diluted with PBS to a final concentration of 5 µg mL^−1^, using 100 µL of such solution per individual, corresponding to 0.5 µg of cHH. The amount of the cHH injected into each crayfish was set from the results of a preliminary experiment showing that the injection of 0.5 µg of cHH determined a significant increase of the glycemic level in the hemolymph. Injections were made through the dorsal abdo-cephalothoracic membrane into the pericardial sinus using a 25 gauge × 5/8′ needle fitted to a 1 mL syringe.

Treated crayfish were left isolated and undisturbed for 30 min. Differently from serotonin and octopamine [13] [19], crayfish did not exhibit any rigid posture immediately after cHH injections.

#### Phase 4: Fighting bouts after injections (T1, T2, T3)

The original pairs were reconstituted and observed following the same procedures as T0. After 10-min acclimatization, the divider was removed and crayfish behaviour was video-recorded for three fighting bouts in sequence of 20 min each (T1, T2, T3). The experiment was timed to record the possible behavioural alterations due to cHH injections as a consequence of a major glucose release expected to occur in T2. In fact, from the literature (e.g. [34]) we know that cHH determines increased glycemia about 1 h after the injection.

Videotapes were then blindly analysed by an unbiased observer (a PhD student), who was well experienced in crayfish behaviour but unaware of the experimental design and predictions. During T0 and the three fighting bouts we recorded:

1. The number and total duration (in s) of fights. A fight began when one opponent approached the other and ended when one of the two individuals ran away, backed off or tail flipped away from the other for at least 10 s at a distance longer than one body length [41]. Tail flipping away is the typical backward swimming response of crayfish. From these parameters we computed the mean duration of fight (in s);
2. Percentage of dominance (%). The number of fights won by an individual as a percentage over the total fights battled. The winner was the individual that did not retreat or that retreated after the opponent had assumed a body down posture or remained motionless. As in familiarization, the alpha was the individual that won more than 60% of the fights battled [40];
3. Fight intensity (measured as the mode of the totalized scores). To each fight, classified as avoidance, threat, week and strong physical interactions, and unrestrained fights (as modified from [4]), a score was assigned from 1 (avoidance) to 5 (unrestrained fight);
4. The fights started by alphas;
5. The time spent motionless (in s).

#### Phase 5: Hemolymph sampling and determination of the final glycemia

To compare variations of glycemia among treatments, immediately after the end of the experiment about 50 µL of hemolymph was drawn from each crayfish from 1300 to 1400 h, as described above.

### Statistical Analyses

Data were first checked for normality and homogeneity of variance using the Kolmogorov-Smirnov and Levene tests, respectively. All data met the assumptions for the parametric tests and were thus analysed accordingly. To test the effect of cHH injections on glycemia, we first computed the difference between glycemic levels after and before the injection, and then we applied a one-way ANOVA (statistic: F), in which the three treatments (CP, RP, and IP) and the two ranks (alpha and beta) were the between-subject factors and the difference in glycemic levels was the variable. The difference among treatments was explored by Tukey *post hoc* tests; in the case of significance, a comparison between alphas and betas was made by independent samples Student’s t test (statistic: t). To analyse the effect of cHH injections on the agonistic behaviour, we applied General Linear Models for repeated measures (GLMs, statistic: F), followed by Tukey *post hoc* tests, where the three treatments (CP, RP, and IP) were between-subject factors and the four fighting bouts (T0, T1, T2, T3) were within-subject factors [42]. If the difference among bouts was significant, we applied one-way ANOVAs (statistic: F) to determine which treatments differed significantly. The level of significance is α = 0.05. The text reports means ± SE.

### Ethical Note

The experiments comply with the current laws of Italy, the country in which they were done. No specific permits were required for the described field studies that did not involve endangered or protected species. The collection of animals did not affect the population density. Individuals were maintained in appropriate laboratory conditions to guarantee their welfare and responsiveness. After the experiments were completed, crayfish were killed by hypothermia because law forbids the release of invasive species into natural water bodies (L.R. 7/2005).

## Results

### Effect of Native cHH on Glycemia (Fig. 3)

As expected, the injection of cHH significantly increased glycemic levels in the crayfish hemolymph (F = 32.874, df = 2,52, P = 0.0001), independently of the hierarchical status of treated individuals (F = 0.0001, df = 1,52, P = 0.996). In fact, after cHH injections, glycemia significantly increased in a similar way (t = 0.57, df = 16, P = 0.995) in both the alphas of RP (t = −10.320, df = 16, P = 0.0001) and the betas of IP (t = 7.668, df = 16, P = 0.0001). Glycemic levels also increased in CP (in alphas: 17.6±6.3 mg dL^−1^, in betas: 16.6±3.2 mg dL^−1^) in response to fighting, but the recorded increment in the crayfish treated with cHH was about 10 times higher (alpha in RP: 178.6±20.4 mg dL^−1^; beta in IP: 177.1±15.0 mg dL^−1^).

**Figure 3:**
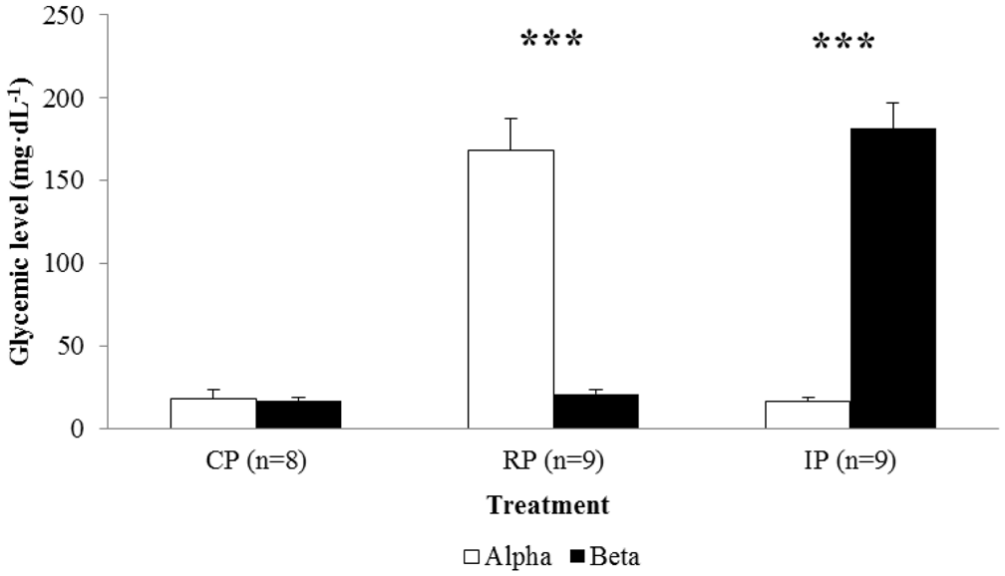
Difference in glycemic levels after (1300–1400 h) and before (0800–0900 h) cHH injections (Mean+SE) in Control Pairs (CP), Reinforced Pairs (RP), and Inverted Pairs (IP), and in alphas (white bars) and betas (black bars). Three asterisks denote significant differences at P<0.001 after Student's t-tests.

### Effect of Native cHH on Agonistic Behaviour and Dominance

**Figure 4:**
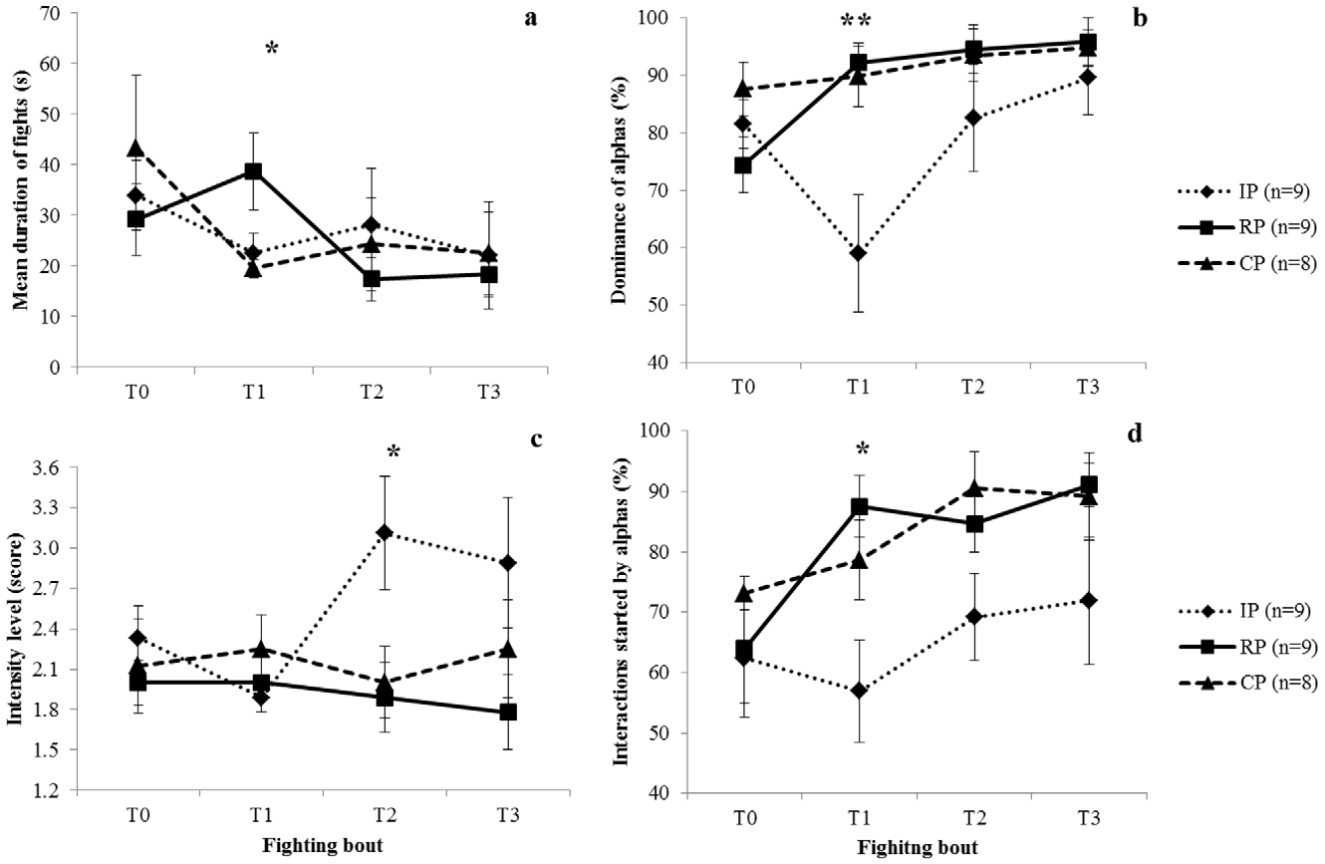
Behavioural parameters across fighting bouts (T0, T1, T2, T3) in Control Pairs (CP), Reinforced Pairs (RP), and Inverted Pairs (IP). Before T0, the initial glycemia was determined; during T0, alpha and beta crayfish were assessed; between T0 and T1, crayfish were subject to the injection of either PBS solution (both alphas and betas in CP, alphas in IP, and betas in RP) or cHH solution (betas in IP and alphas in RP); from T1 to T3, crayfish behaviour was recorded and, then, the final glycemia was determined. Means (± SE) of: (a) duration of fights; (b) percentage of dominance; (c) fight intensity level; (d) number of fights started by alphas. One and two asterisks denote significant difference at P<0.05 and P<0.01, respectively, after one-way ANOVAs.

**Figure 5:**
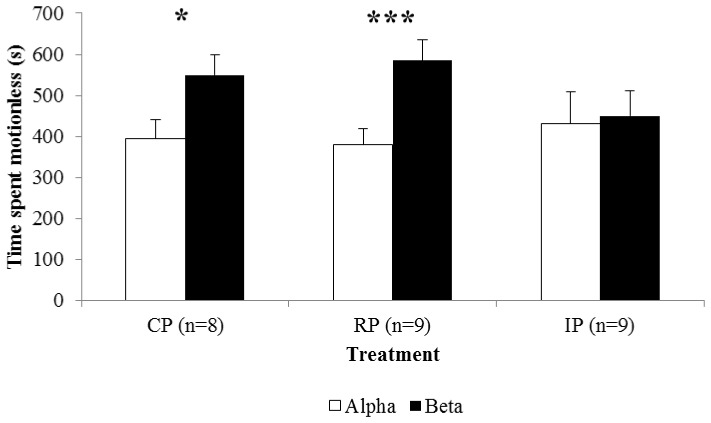
Mean time spent motionless (+SE) in Control Pairs (CP), Reinforced Pairs (RP) and Inverted Pairs (IP), and in alphas (white bars) and betas (black bars). One and three asterisks denote significant differences at P<0.05 and P<0.001, respectively, after Student’s t-tests.

The total duration (F = 11.414, df = 3,69, P = 0.0001) and the number (F = 7.061, df = 3,69, P = 0.0001) of fights tended to decrease from T1 to T3 without any difference among treatments (F = 0.356, df = 2,23, P = 0.704 and F = 9.598, df = 6, 69, P = 0.748, respectively). As a consequence, the mean duration of fights was progressively shorter (F = 3.166, df = 3,69, P = 0.032) even if, immediately after the cHH injection, RP pairs combated longer than CP (F = 3.982, df = 2,25, P = 0.033; IP = CP<RP) (Fig. 4a). Alphas increased dominance across fighting bouts (F = 7.817, df = 3,69, P = 0.0001) independently of the treatment (F = 5.111, df = 6,69, P = 0.0001). The increase in the agonistic behaviour of betas immediately after the cHH injection reduced the dominance of alphas in IP (F = 7.058, df = 2,69, P = 0.004; IP<RP = CP), leading to a temporary reversal of hierarchy in T1 (Fig. 4b). Crustacean HH injections also affected the intensity of fights (F = 3.536, df = 2,23, P = 0.046). In particular, beginning from T2, IP pairs interacted stronger than the other pairs (F = 4.281, df = 2,25, P = 0.026; CP = RP<IP), with fight intensity remaining high until the end of the experiment (Fig. 4c). More often, fights were started by the alphas (F = 5.765, df = 3,69, P = 0.001), although a difference was found with treatments (F = 3.571, df = 2,23, P = 0.045). While no difference was found between CP and RP, in IP the number of fights that the initial alpha started significantly decreased in T1 (F = 5.371, df = 2,25, P = 0.012; IP<CP = RP), as a consequence of cHH injections on betas. Alphas started a lower number of fights than betas also in T2 (F = 3.285, df = 2,25, P = 0.056) but this number tended to increase progressively through the experiment (F = 1.939, df = 2,25, P = 0.167) (Fig. 4d). Overall the time spent motionless increased with time (F = 2.992, df = 3,92, P = 0.035), with betas remaining motionless for longer than alphas (t = −2.355, df = 31, P = 0.025). The treatment had also an effect on activity (F = 3.594, df = 2,92, P = 0.031): cHH injections induced a more intense locomotion, so treated betas in IP spent the same time motionless as alphas (t = −0.355, df = 35, P = 0.725), whereas the different activity between treated alphas in RP and the respective beta increased (t = −4.797, df = 35, P = 0.0001) (Fig. 5).

## Discussion

Our study analysed the effects of cHH on the agonistic behaviour of crayfish and demonstrated, for the first time, its role in enhancing it, up to reverse, although transitorily, the rank. The supporting evidence is: as expected, (1) in CP and RP alphas increased dominance. On the contrary, (2) in IP, betas became likely to initiate and escalate fights and, consequently, increased dominance till a temporary reversal of the hierarchy, but the original rank was re-established. (3) In comparison with control individuals, fights of treated alphas were longer and reached a higher intensity in treated betas. (4) IP betas showed reduced time spent motionless. To summarize, independently of prior social experience, cHH injections induced expression of dominance that differs in relation to the original rank of the individual. These behavioural changes were associated not only with an increased glycemia in the crayfish hemolymph, as well known in the literature [34], but also with the reduced time spent motionless. Our results here are consistent with what was previously described for exogenous serotonin on *P. clarkii* [19] and other crustaceans, including *H. americanus* [13], *A. astacus* [20]–[21], and *M. quadrispina* [18]. Serotonin, in fact, elicits the occurrence of dominant postures and aggression, in terms of the number of fights of high intensity executed, and determines a temporary reversal of hierarchies. However, serotonin shows a longer lasting effect than cHH: the original hierarchy in *P. clarkii* was reconstituted 1 h after the injection of serotonin [19] but only 30 min after the injection of cHH.

Our results show that cHH can modulate the neurons controlling the direct expression of agonistic behaviour also mobilizing the energetic stores needed for the increased fighting activity. Natural fluctuations of cHH release seem to be regulated by changes in central neuromodulation due to environmental and/or endogenous influences [43]. Several neurotransmitters and neuropeptides are involved on cHH release. Serotonin is recognized to play an important role in mediating the release of cHH [44]–[45]: serotonin injection is followed by a release of cHH that causes hyperglycemia [46]–[47] [22]. As a further confirmation of the occurrence of the serotonin-cHH-glycaemia physiological axis, immunoreactive and ultrastructural studies have demonstrated serotonergic synaptic structures on the axonal ramification of the cHH-producing cells of the X-organ of crayfish [48], *P. clarkii* included [49]. The involvement of the serotonin-cHH-glycemia physiological axis could explain both the mechanisms through which cHH controls agonism and the expression and timing of dominant behaviours triggered by either cHH or serotonin injections. The availability of an adequate amount of cHH by synthesising it with the correct post-translational modifications conferring a full biological activity [50] will allow further validation or rejection of this hypothesis.

Consistent with the study on the serotonin effects on *P. clarkii* [19], also the cHH did not lead to a permanent inversion of the dominance hierarchy. Cheating seems not to be sufficient to maintain the role of dominant in prolonged fights against stronger opponents. Intrinsic properties of crayfish other than body size, weight, chelar dimensions or circulating neuropeptides may likely determine the structure of dominance hierarchies in decapods. For instance, in the American lobster, *H. americanus*, the outcome of contests between size-matched individuals was predicted from hidden cues such as plasma protein level and exoskeleton calcium concentration [51]. These variables are not clearly visible to the rivals, but fighting lobsters may indirectly assess them by claw contraction forces, the resistance of the exoskeleton to pressure, and general fighting vigour [51]. Notwithstanding the neuropeptides injected, betas have neither the physical characteristics nor the experience of a dominant, and prolonged fights could result in both losing time/energy and increasing the risks of injury that eventually may lead to their death [52]. The original rank is thus quickly re-established since it allows betas to minimize the costs and risks of fighting with a superior individual. As a consequence, the relevance of both intrinsic physical characteristics and experience cannot be excluded in the dynamics of dominance hierarchies.

Undoubtedly, behavioural physiology opens new avenues for our understanding of the functioning of cHH and is expected to unravel its role in modulating invertebrate agonistic behaviour. Future researches are obviously needed to answer the exciting questions of how physiology and environment interact in regulating the neural systems underlying the formation and maintenance of social hierarchies across species.
